# Enhancing Substance Use Detection in Clinical Notes with Large Language Models

**DOI:** 10.21203/rs.3.rs-6615981/v1

**Published:** 2025-05-15

**Authors:** Fabrice Harel-Canada, Anabel Salimian, Brandon Moghanian, Sarah Clingan, Allan Nguyen, Tucker Avra, Michelle Poimboeuf, Ruby Romero, Arthur Funnell, Panayiotis Petousis, Michael Shin, Nanyun Peng, Chelsea L. Shover, David Goodman-Meza

**Affiliations:** a Computer Science Department, University of California, Los Angeles, 404 Westwood Plaza Suite 277, Los Angeles, 90095, CA, USA; b Semel Institute for Neuroscience and Human Behavior at University of California, Los Angeles, 760 Westwood Plaza, Los Angeles, 90024, CA, USA; c University of California, Los Angeles, 200 Medical Plaza Suite 365C, Los Angeles, 90024, CA, USA; d Integrated Substance Abuse Programs at University of California, Los Angeles, 10911 Weyburn Ave, Ste. 200, Los Angeles, 90024, CA, USA; e David Geffen School of Medicine at University of California, Los Angeles, 10833 Le Conte Ave, Los Angeles, 90095, CA, USA; f Division of General Internal Medicine and Health Services Research, University of California, Los Angeles, 1100 Glendon Ave STE 850, Los Angeles, 90024, CA, USA; g Clinical and Translational Science Institute, University of California, Los Angeles, 924 Westwood Blvd Suite 420, Los Angeles, 90024, CA, USA; h Department of Geography, University of California, Los Angeles, 1255 Bunche Hall, Los Angeles, 90095, CA, USA; i Kirby Institute, University of New South Wales, Wallace Wurth Building (C27), Cnr High St & Botany St, UNSW, Sydney, 2052, NSW, Australia

**Keywords:** NLP, natural language processing, substance use, drug use, people who inject drugs

## Abstract

Identifying substance use behaviors in electronic health records (EHRs) is challenging because critical details are often buried in unstructured notes that use varied terminology and negation, requiring careful contextual interpretation to distinguish relevant use from historical mentions or denials. Using MIMIC-III/IV discharge summaries, we created a large, annotated drug detection dataset to tackle this problem and support future systemic substance use surveillance. We then investigated the performance of multiple large language models (LLMs) for detecting eight substance use categories within this data. Evaluating models in zero-shot, few-shot, and fine-tuning configurations, we found that a fine-tuned model, Llama-DrugDetector-70B, outperformed others. It achieved near-perfect F1-scores (≥ 0.95) for most individual substances and strong scores for more complex tasks like prescription opioid misuse (F1=0.815) and polysubstance use (F1=0.917). These findings demonstrate that LLMs significantly enhance detection, showing promise for clinical decision support and research, although further work on scalability is warranted.

## 1. Introduction

Identifying persons who use drugs and understanding their related behaviors are critical for improving patient care. In electronic health records (EHRs), the detailed nuances of substance use are primarily documented within free-text notes, a domain of knowledge confined mainly to the direct care providers who interact with patients daily [1, 2]. While EHRs contain a wealth of data [3], this crucial information regarding substance use and related issues presents a significant challenge for researchers, hospital administrators, and public health agencies seeking to monitor broader usage trends and inform policy [4, 5]. The current landscape often leaves these higher-level stakeholders operating without a comprehensive, aggregated view of substance use patterns within their populations.

Natural language processing (NLP) offers a promising solution to bridge this gap by extracting actionable insights from the vast amounts of unstructured text data in EHRs [6, 7, 8]. As a subfield of artificial intelligence, NLP focuses on developing algorithms to understand and analyze human language [9]. These techniques have been applied to various tasks, such as classifying clinical notes, extracting patient information [10], and screening for potential future substance use [11]. In the domain of substance use disorders, NLP has demonstrated effectiveness in detecting opioid misuse [12, 13, 14], identifying people who inject drugs (PWID) [15], and recognizing substances involved in overdoses [16]. By transforming these detailed clinical notes into analyzable data, NLP could be used to monitor usage trends, allocate resources effectively, and develop targeted interventions for at-risk individuals.

Recent advances in NLP have led to the development of two prominent types of models: BERT-style encoders [17, 18, 19, 20] and GPT-style decoders [21, 22, 23], which have significantly improved our ability to process and generate human language [24]. These large language models (LLMs), extensively pre-trained on vast text datasets, can perform a wide range of tasks, often with little (few-shot) to no (zero-shot) task-specific data. This flexibility makes them particularly attractive for scenarios where labeled data is scarce, such as in clinical domains focused on substance use [25, 26, 27, 28, 29]. Despite their proven value, their applications to substance use detection in unstructured EHRs are under-explored. Therefore, this study aimed to evaluate the performance of contemporary zero-shot and few-shot NLP models in identifying substance use and related features from unstructured text in EHRs.

## 2. Methods

### 2.1. Dataset

We performed a retrospective study to evaluate the performance of different LLMs at identifying reported substances used by patients within unstructured text from EHRs. As this analysis involved only de-identified, publicly available data, the University of California, Los Angeles Institutional Review Board (IRB) determined this study to be exempt from IRB oversight. We used the MIMIC dataset, a comprehensive publicly available repository of de-identified EHRs from patients admitted to Beth Israel Deaconess Medical Center. We included records from both MIMIC-III (2001–2012) [30] and MIMIC-IV (2008–2019) [31].

### 2.2. Substance Classes

Given the prevalence of polysubstance use, we framed this task as a multi-label text classification problem to capture concurrent use. In our setup, the input was a medical note containing potential references to substance use. The output was a binary vector indicating the presence or absence of eight items of interest: heroin, cocaine, methamphetamine, illicit use of prescription opioids and benzodiazepines, cannabis, injection drug use (IDU), and general drug use (Any). Although fentanyl was also considered an item of interest, it was excluded from the final set due to its infrequency in our dataset.

#### 2.2.1. Human Annotation

We identified 1,151 notes containing keywords relevant to the eight drug classes. Five team members (AS, BM, SC, AN, TA) were trained to recognize both explicit and nuanced mentions of substances used based on a pre-specified annotator guide (Appendix A). For instance, while prescription opioids were frequently mentioned benignly in medical notes, identifying illicit use required careful contextual understanding. Annotators highlighted spans of one or more words and classified them under one of the drug classes. Each text span was assigned a single drug class, although multiple spans could be annotated within the same sentence or note.

All team members annotated the same set of 100 notes (10%), and kappa statistics [32] were computed to assess inter-annotator agreement. Upon achieving a kappa score above 0.80 for each class, indicating strong agreement [33], annotators proceeded to single-annotate a subset of the remaining notes. A final team member (AS) then reviewed all annotations for accuracy.

#### 2.2.2. Data Preprocessing

Due to the significant length of the original medical notes, which posed challenges for standard NLP techniques, we employed span-level annotations. This method breaks down the text into meaningful segments (spans) for individual analysis. While this initially allowed consideration of token classification models—assigning classes like drug names to each word [34, 35], the potential computational intensity of classifying every token led us to adopt a different strategy. We reframed the task as multi-label sentence classification, assigning multiple relevant labels (e.g., identifying both “cocaine” and “cannabis”) to each sentence, thereby capturing diverse information more efficiently than word-by-word analysis.

By tokenizing the annotated medical notes into sentences, we compiled a dataset of 274,602 rows, with only 3,948 containing drug mentions. To evaluate zero and few-shot model performance, we created class-balanced dataset splits for training, validation, and test splits with 10%:10%:80% data points, respectively. We distributed all instances of these classes across the dataset splits. However, some skew was inevitable due to the prevalence of these substances. For example, heroin and cocaine mentions were more common and are overrepresented relative to other drug classes, such as methamphetamine.

### 2.3. Detection Models

We evaluated a range of NLP models for detecting substance use in medical notes. We considered key dimensions such as model architecture, pretraining specialization, and availability in a comprehensive, concurrent analysis. We compared smaller, more efficient BERT-style encoders with larger GPT-style decoders (commonly known LLMs), each offering distinct benefits in terms of processing speed, computational demands, and task performance. BERT-style encoders capture context bidirectionally, making them effective for understanding nuanced medical language, while GPT-style decoders process text sequentially, optimizing for fluent generation but lacking full bidirectional context.

We also assessed the impact of domain-specific pre-training, particularly in the medical field, to determine whether specialized training enhances the models’ ability to detect substance use accurately. Additionally, our selection included both open-source and proprietary models to address critical concerns like cost, accessibility, transparency, and privacy—factors that are especially important in medical applications. To further explore the impact of few-shot fine-tuning, we created Llama-DrugDetector in both 8B and 70B parameter versions, optimized for substance use identification tasks in electronic health records using only a limited number of examples (*n* = 804). A summary of the models we studied is provided in Table 1, with additional details on each model available in Appendix C, and a description of our fine-tuning process in Appendix D.

### 2.4. Detection Pipelines

We developed custom detection pipelines to evaluate the performance of various model paradigms in detecting reported substances used and IDU.

For the BERT-style encoders, zero-shot analysis was not advisable because the added classification layers require at least some tuning to map inputs to outputs meaningfully. Therefore, we focused on few-shot fine-tuning, both with and without additional medical domain pre-training. This process involves updating the model weights based on errors observed in a small set of examples.

In addition to few-shot fine-tuning, LLMs support in-context learning (ICL) [41]. In this setting, the LLMs learn the task directly from a few examples provided within the context of the prompt, without requiring updates to the model itself. We evaluated the LLMs under zero-shot (no examples) and few-shot (few examples) configurations randomly drawn from the validation split of the dataset. This allowed us to characterize the performance of few-shot fine-tuning combined with few-shot ICL. For locally hosted LLMs, we implemented our prompting pipelines using guidance [42], a constrained decoding framework that enforces well-formed outputs. Since guidance requires access to token probabilities to function optimally and proprietary LLM providers do not provide this data, we instead implemented a separate pipeline for the GPT family of models in langchain [43].

### 2.5. Evaluation Metrics

We compared the performance of all models to identify the best combinations of fine-tuning and prompting strategies. Using the held-out test split (n = 6443) of the DrugDetection dataset, we calculated diagnostic metrics including F1-score, accuracy, sensitivity (i.e., recall), positive predictive value (i.e., precision), specificity, and negative predictive value. The F1-score, which balances positive predictive value and sensitivity, is particularly useful in cases with an uneven distribution of positive and negative instances.^2^ We calculated 95% confidence intervals (CIs) via bootstrapped resampling. We bootstrapped the testing set with replacement 1000 times, running each test on 100 samples and calculating diagnostic metrics for each resample. The 2.5th and 97.5th percentiles were reported as the lower and upper ends of the CI, respectively, and the 50th percentile as the mean. Lastly, we performed a manual error analysis of the false-positive and false-negative predictions from the best-performing NLP model. All statistical analyses were performed using Python 3.12 software.

### 2.6. Error Analysis

Lastly, we conducted several rounds of error analysis to identify specific weaknesses in model performance, categorizing and quantifying the most common errors. Based on these analyses, we iteratively refined our prompts to address these issues. The final prompt template we used for all LLMs is available in Appendix E.

## 3. Results

For simplicity of presentation, we focus on overall performance by model aggregated across all drug classes. Complete performance breakdowns by metric can be found in the Appendix F.

### 3.1. Dataset Statistics

The text inputs in the DrugDetection dataset average 17.2 words each. These were derived from the original notes, which, prior to sentence tokenization, were substantially longer, averaging 239 sentences and about 2840 words per note.

Table 2 summarizes key statistics for each dataset split and for the full DrugDetection dataset. To better evaluate the precision of substance detection systems, we included medical notes unrelated to substance use (see the “None” column), making up roughly 50% of the overall dataset. Additional details, including substance co-occurrence patterns, are provided in Appendix B.

### 3.2. BERT-style Model Evaluation

Table 3 compares four BERT-style decoder models on our DrugDetection dataset, including the general-purpose bert-base-uncased and three bio-clinical variants. Contrary to conventional expectations, the base model demonstrated competitive performance, achieving the highest accuracy (0.691, 95% CI: 0.679–0.701) and specificity (0.962, 95% CI: 0.959–0.964) among all models, along with superior precision (0.334, 95% CI: 0.290–0.378). While ClinicalBERT attained the highest F1-score (0.308, 95% CI: 0.298–0.318) and sensitivity (0.363, 95% CI: 0.356–0.371), its performance margins over the base model remain narrow, with overlapping confidence intervals in most metrics. The bio-clinical models showed mixed results: Bio\_ClinicalBERT achieved marginally better accuracy (0.689 vs. 0.683) than ClinicalBERT but lower sensitivity, while biobert-v1.1 in F1-score (0.276) and precision (0.265). All models exhibit strong negative predictive value (NPV ≥ 0.966) and specificity (≥ 0.954), indicating robust identification of true negatives, but struggle with positive case detection (sensitivity ≤ 0.363).

### 3.3. Zero-shot LLM Model Evaluation

Table 4 compares zero-shot performance of Llama-3 variants with and without bio-clinical adaptation. The base Llama-3.1–8B-Instruct maintained superior performance among 8B models, achieving the highest F1-score (0.706) and accuracy (0.716), though domain-adapted Llama3-OpenBioLLM-8B demonstrated exceptional precision (0.728) and specificity (0.987). Notably, Llama3-Med42–8B showed dramatic sensitivity (0.968) at the cost of precision (0.499), suggesting over-detection tendencies. For 70B models, the generalist DeepSeek-R1-Distill-Llama-70B remained dominant with peak F1-score (0.871) and accuracy (0.860). All models exhibited strong negative predictive value (NPV ≥ 0.946) and specificity (≥0.833), mirroring patterns observed in BERT-style detectors (Table 3), but LLMs demonstrated substantially higher sensitivity (recall ≥0.680 vs. ≤0.363 in BERT models). While domain adaptation showed potential in specific metrics (*e.g*., Llama3-OpenBioLLM-8B’s precision outperformed base models by 15.8%), the general superiority of base architectures motivated our selection of DeepSeek-R1-Distill-Llama for few-shot fine-tuning.^3^

### 3.4. Few-Shot Fine-Tuning and Few-Shot ICL

**Figure 1:**
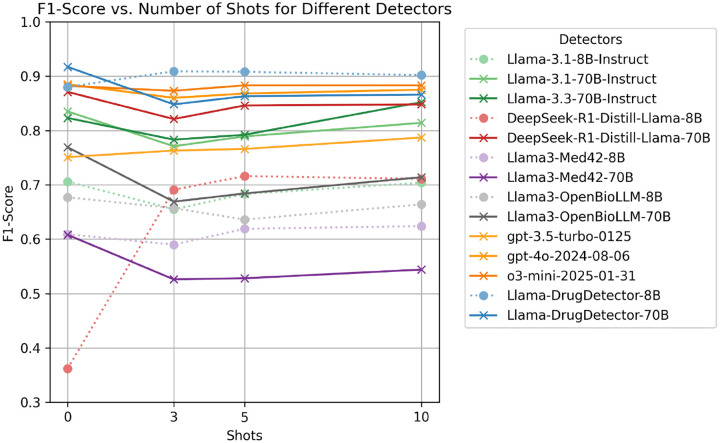
Overall F1-score per model when given N-Shot examples in the prompt, with models grouped by family and colored accordingly. Dotted lines indicate 8B models, whereas solid lines denote 70B or models of unspecified size. Across detectors, the maximum improvement in F1-Score was 35.4% (DeepSeek-R1-Distill-Llama-8B), and the average improvement was ~ 1.3%.

Figure 1 illustrates the impact of incorporating few-shot examples within the context of the prompt before the main classification task. In 6 out of 15 instances, including few-shot examples led to an enhancement in F1-score compared to the zero-shot baseline. Notably, DeepSeek-R1-Distill-Llama-8B exhibited a substantial 35.4% improvement. On average, few-shot incontext learning resulted in a ~ 1.3% performance boost. Additionally, we observed that combining few-shot fine-tuning with few-shot ICL yielded significant benefits. Our best overall model, Llama-DrugDetector-70B, achieved the highest zero-shot performance (91.9%) but did not benefit from few-shot examples. However, Llama-DrugDetector-8B did benefit from few-shot examples and was the highest performing 8B model. This demonstrated the potential of few-shot ICL in enhancing model accuracy, particularly when integrated with fine-tuning strategies.

### 3.5. Comparing Best Overall Performance

Table 5 compares model performance in the challenging polysubstance detection setting, where models must simultaneously identify all relevant drug classes (accuracy requires perfect multi-label classification). Our fine-tuned Llama-DrugDetector-70B achieved the highest F1-score (0.917), while its 8B counterpart (Llama-DrugDetector-8B) demonstrated exceptional accuracy (0.939) and specificity (0.994), suggesting particular strength in avoiding false positives across multiple substance categories. Proprietary models without fine-tuning show mixed performance: o3-mini-2025-01-31 achieved peak specificity (0.994) and competitive precision (0.893) but trailed in F1-score (0.883 vs. 0.917). The all-classes-correct requirement exacerbated architectural disparities, with 70B LLMs achieving 2.97× higher F1-scores than the best-performing BERT-style variants (0.917 vs. 0.308). Finally, fine-tuned open-source models consistently outperformed proprietary counterparts in critical metrics – Llama-DrugDetector-70B surpassed gpt-4o-2024-08-06 in F1-score (0.917 vs. 0.885).

### 3.6. Comparing Substance-Specific Performance

The F1 performance for detecting substances individually is presented in Table 6, with similar tables for other metrics available in Appendix F. Detection complexity and performance varies substantially by substance class, and different models possess different strengths. For example, prescription opioid misuse proved most challenging (best F1: 0.830, Llama-DrugDetector-8B), while all other classes exhibited perfect or near-perfect detection (*geq* 0.95). Heroin detection peaked with o3-mini-2025-01-31 (0.985), while cocaine identification was strongest in Llama-DrugDetector-70B (0.994). Methamphetamine detection reached perfection (F1: 1.000) in Llama-3.3-70B-Instruct, though its narrow applicability is evident from lower scores in prescription opioid misuse (0.655).^4^

### 3.7. Error Analysis

Table 7 presents the final error analysis for the best-performing model, our fine-tuned DrugDetector-70B. The most common issue was insufficient evidence, where the model made assumptions unsupported by the text – such as inferring heroin use from injection drug use (n = 126) or assuming illicit use of prescription opioids (n = 103). Another major category was simple failures to identify substances (n = 113), including missed mentions or negated statements. Additional errors stemmed from confusion, such as hallucinating drug use (n = 30), misattributing substance use to the patient rather than a family member, or misreading typos.

## 4. Discussion

This study rigorously evaluated the efficacy of various NLP models, ranging from traditional BERT-style encoders to decoder-only LLMs, in the critical task of detecting substance use mentions within electronic health records. Our findings demonstrate a paradigm shift in the potential of NLP for this domain, revealing that even with limited fine-tuning data, contemporary LLMs can achieve remarkable diagnostic performance, significantly surpassing previous benchmarks that relied on extensive training datasets [15, 14, 13]. Notably, our open-source model fine-tuned using a few hundred training examples, Llama-DrugDetector-70B^5^, achieved an F1-score of 0.919 for concurrent polysubstance use, while substance-specific F1 scores ranged from 0.815 for prescription opioid misuse to 0.994 for cocaine use. This exceptional performance, coupled with tighter confidence intervals compared to proprietary models like gpt-4o-2024-08-06, underscores the potential for enhanced clinical reliability and the feasibility of deploying such sophisticated tools in real-world healthcare settings. The ability of our model to identify both explicit and contextually nuanced substance references addresses a significant limitation of earlier rule-based systems [13], offering a more adaptable solution to the evolving landscape of clinical documentation.

A consistent trend throughout our analysis was the superior performance of LLMs over BERT-style encoders across a spectrum of metrics, particularly in the nuanced task of substance-specific detection. This advantage likely stems from the inherent capacity of LLMs to model complex language patterns and capture subtle contextual cues, which are crucial for accurately identifying drug use within clinical narratives [44, 45]. The observation that open-source LLMs often matched or even exceeded the performance of proprietary models like GPT-4o has significant implications for accessibility and deployment. Importantly, open-source models offer the advantage of being locally hosted, making them more suitable for production use in medical and clinical settings where data privacy is legally and ethically mandated [46].

Beyond the immediate clinical applications of improved drug detection, our findings have significant implications for public health surveillance and research. The ability to accurately and efficiently extract information about substance use from EHRs can provide valuable data for monitoring trends in drug use prevalence, which are often difficult to ascertain through traditional methods [47, 48, 49, 50]. By transforming unstructured clinical text into actionable data, our approach can facilitate a more comprehensive understanding of the evolving drug landscape and enable better-informed public health interventions and policy decisions at both the hospital-level and government-level. The potential for integrating insights from our models with other public health data sources, such as overdose statistics, could further enhance our ability to track and respond to the opioid crisis and other substance use challenges.

The error analysis conducted on our best-performing model, Llama-DrugDetector-70B, provided valuable insights into its remaining weaknesses. The prevalence of errors related to insufficient evidence, failures to detect substances, and confusion between similar terms highlights the challenges inherent in interpreting complex medical language and the need for further refinement in handling contextual nuances. These findings suggest that future work could focus on improving the model’s ability to reason about implicit information, handle negation and subtle linguistic cues, and better distinguish between substances with similar names. Techniques such as incorporating more sophisticated prompt engineering strategies or augmenting the fine-tuning data with examples specifically designed to address these error types might also be beneficial.

## 5. Limitations

This study has several important limitations. First, the reliance on data from a single medical center in Boston, spanning the years 2001–2019, may limit the generalizability of our findings. Drug use patterns have evolved over time, particularly with the shift from heroin to fentanyl and other synthetic drugs. Prior work [51, 52, 53] has shown that different regions of the US exhibit unique drug use profiles that continue to change. Second, we focused on analyzing short medical notes extracted from larger patient profiles. While this approach enabled us to target specific instances of drug-related language, it also introduced the possibility of losing critical context during sentence tokenization. The potential loss of context could result in the misinterpretation of drug-related language, particularly in complex cases where substance use is inferred from patient history rather than explicitly stated. Other sources of medical documentation could have been relevant to inferring drug use, such as lab results or other provider notes (e.g., social worker or nusring notes). Third, while LLMs offer superior performance in terms of accuracy and language understanding, they demand significantly more computational resources. For instance, BERT-style models process each input in approximately 0.002 seconds, whereas LLMs require between 0.3 to 20 seconds, depending on the model. This disparity becomes a substantial challenge when scaling up to process entire patient notes, which contained an average of 239 sentences in our dataset. Expanding the input size to encompass full patient profiles would necessitate advancements in processing speed for LLMs to make such an approach more viable in real-world applications. The significant computational demands of LLMs, contrasted with the faster but more limited BERT-style models, present a clear trade-off between accuracy and efficiency. One potential solution is to use LLM quantization techniques [54], which reduce model size, trading off some accuracy for increased speed. Future research should explore hybrid models that combine the contextual depth of LLMs with the efficiency of smaller models, potentially through innovative architectures or the application of LLM quantization techniques.

## 6. Conclusions

This study establishes that modern LLMs, when combined with few-shot fine-tuning, achieve near-perfect performance in detecting substance use within clinical narratives – surpassing both traditional NLP approaches and proprietary models. Our open-source Llama-DrugDetector-70B attained near-perfect accuracy for most substance classes (except prescription opioid misuse) using only a few hundred training examples, demonstrating that domain-specific performance no longer requires massive labeled datasets or restrictive proprietary systems. These advances carry immediate practical implications: Hospital administrators could deploy such models to flag systemic substance use risks in real time, while public health agencies might leverage them to detect emerging drug trends from unstructured EHR data. However, some challenges remain in minimizing over-interpretation errors (e.g., inferring heroin use from syringe mentions) and optimizing computational costs for clinical workflows. Future work should expand to emerging substances like synthetic opioids (e.g. fentanyl) and explore federated learning frameworks to enhance generalizability across healthcare systems. By open-sourcing our models and benchmarks, we aim to catalyze community-driven improvements in this critical area of clinical NLP.

## Figures and Tables

**Table 1:** Summary of selected NLP models categorized by architecture type (Arch.), specialization, and availability.

Model Name	Arch.	Specialization	Availability
BERT [17]	Encoder	Generalist	Open Source
BioBERT [18]	Encoder	Biomedical	Open Source
ClinicalBERT [20]	Encoder	Clinical	Open Source
Bio_ClinicalBERT [19]	Encoder	Biomedical & Clinical	Open Source
GPT-4o [36]	Decoder	Generalist	Proprietary
Llama-3-Instruct [23]	Decoder	Generalist	Open Source
Llama-3.1-Instruct [23]	Decoder	Generalist	Open Source
Llama-3.3-Instruct [23]	Decoder	Generalist	Open Source
DeepSeek-R1-Distill-Llama [37]	Decoder	Generalist	Open Source
MedLlama3 [38]	Decoder	Biomedical*	Open Source
Llama3-OpenBioLLM [39]	Decoder	Biomedical*	Open Source
Llama3-Med42 [40]	Decoder	Clinical*	Open Source
Llama-DrugDetector (Ours)	Decoder	Substance Use	Open Source

Where possible, we study different sizes of the same model (e.g., Llama-3-Instruct comes in versions with 8B or 70B parameters). Asterisks (*) indicate reported domain, but the authors have released no official datasets to confirm content. Llama-DrugDetector is our fine-tuned version of DeepSeek-R1-Distill-Llama.

**Table 2:** Counts of each drug class by split for the DrugDetection dataset. Abbreviations: Methamphetamine Use (Meth.), Benzodiazepine Use (Benzo.), Prescription Opioids Misuse (Rx. Opiods), Injection Drug Use (IDU), and General Drug Use (Any). Total shows the total number of medical notes with zero-to-many substances present in each.

SPLIT	Heroin	Cocaine	Meth.	Benzo.	Rx. Opiods	Cannabis	IDU	Any	None	Total
**TRAIN**	93	65	9	26	13	13	128	402	402	804
**VALIDATION**	94	66	9	26	14	14	128	403	403	806
**TEST**	749	528	72	232	122	121	1041	3143	3300	6443
**TOTAL**	936	659	90	284	149	148	1297	3948	4105	8053

**Table 3:** Performance of BERT-style decoder models with various types of additional pre-training on bio-clinical data. bert-base-uncased is the base model.

Detectors	F1-Score	Accuracy	Sensitivity (Recall)	Positive Predictive Value (Precision)	Negative Predictive Value	Specificity
\texttt{biobert-v1.1}	0.276 (0.271 – 0.281)	0.666 (0.654 – 0.677)	0.329 (0.324 – 0.334)	0.265 (0.240 – 0.300)	0.969 (0.968 – 0.971)	0.954 (0.952 – 0.956)
\texttt{Bio_ClinicalBERT}	0.279 (0.276 – 0.283)	0.689 (0.679 – 0.703)	0.311 (0.307 – 0.315)	0.256 (0.252 – 0.261)	0.966 (0.964 – 0.967)	**0.962** **(0.959 – 0.964)**
\texttt{bert-base-uncased}	0.295 (0.285 – 0.305)	**0.691** **(0.679 – 0.701)**	0.328 (0.322 – 0.335)	**0.334** **(0.290 – 0.378)**	0.969 (0.967 – 0.971)	**0.962** **(0.959 – 0.964)**
\texttt{ClinicalBERT}	**0.308** **(0.298 – 0.318)**	0.683 (0.670 – 0.693)	**0.363** **(0.356 – 0.371)**	0.288 (0.269 – 0.309)	**0.971** **(0.969 – 0.972)**	0.954 (0.952 – 0.957)

**Table 4:** Zero-shot performance metrics of various LLMs with and without additional bio-clinical pre-training, grouped by model size (8B vs. 70B). Llama-3 serves as the base model without targeted domain pre-training. Results indicate that specialized bio-clinical training does not consistently enhance performance for drug detection, motivating the use of a general-purpose model for subsequent task-specific fine-tuning.

Detectors	F1-Score	Accuracy	Sensitivity (Recall)	Positive Predictive Value (Precision)	Negative Predictive Value	Specificity
DeepSeek-R1-Distill-Llama-8B	0.362 (0.347 – 0.374)	0.616 (0.606 – 0.629)	0.753 (0.723 – 0.777)	0.285 (0.274 – 0.296)	0.946 (0.944 – 0.949)	0.833 (0.825 – 0.840)
MedLlama3-8B	0.403 (0.392 – 0.413)	0.101 (0.095 – 0.107)	0.928 (0.914 – 0.940)	0.304 (0.293 – 0.314)	**0.988 (0.985 – 0.992)**	0.628 (0.623 – 0.634)
Llama3-Med42-8B	0.609 (0.586 – 0.628)	0.705 (0.693 – 0.715)	**0.968 (0.961 – 0.975)**	0.499 (0.479 – 0.516)	0.987 (0.986 – 0.988)	0.928 (0.924 – 0.932)
Llama3-OpenBioLLM-8B	0.677 (0.657 – 0.695)	0.700 (0.689 – 0.712)	0.680 (0.659 – 0.700)	**0.728 (0.703 – 0.753)**	0.947 (0.944 – 0.949)	**0.987 (0.986 – 0.988)**
Llama-3.1-8B-Instruct	**0.706 (0.691 – 0.722)**	**0.716 (0.706 – 0.724)**	0.941 (0.930 – 0.952)	0.618 (0.602 – 0.637)	0.979 (0.977 – 0.981)	0.957 (0.955 – 0.959)
Llama3-Med42-70B	0.608 (0.591 – 0.626)	0.661 (0.651 – 0.672)	**0.988 (0.983 – 0.992)**	0.468 (0.451 – 0.485)	0.997 (0.996 – 0.998)	0.908 (0.904 – 0.912)
Llama3-OpenBioLLM-70B	0.769 (0.747 – 0.789)	0.720 (0.710 – 0.730)	0.987 (0.980 – 0.994)	0.659 (0.630 – 0.688)	**0.999 (0.998 – 0.999)**	0.941 (0.938 – 0.944)
Llama-3.3–70B-Instruct	0.823 (0.813 – 0.832)	0.795 (0.785 – 0.804)	0.985 (0.977 – 0.993)	0.746 (0.729 – 0.761)	0.997 (0.997 – 0.998)	0.967 (0.966 – 0.969)
Llama-3.1–70B-Instruct	0.835 (0.823 – 0.849)	0.828 (0.820 – 0.838)	0.978 (0.967 – 0.986)	0.769 (0.751 – 0.787)	0.997 (0.997 – 0.998)	0.972 (0.970 – 0.974)
DeepSeek-R1-Distill-Llama-70B	**0.871 (0.857 – 0.883)**	**0.860 (0.851 – 0.868)**	0.958 (0.948 – 0.970)	**0.831 (0.814 – 0.847)**	0.991 (0.990 – 0.992)	**0.984 (0.983 – 0.985)**

**Table 5:** Performance by metric on the held-out test set (n=6443), demonstrating that our fine-tuned models consistently outperform others across nearly all quality dimensions. Parentheses indicate 95% bootstrapped confidence intervals.

Arch	Detector	Shots	F1-Score	Accuracy	Sensitivity (Recall)	Positive Predictive Value (Precision)	Negative Predictive Value	Specificity
BERT	biobert-vl.1	0	0.276 (0.271 – 0.281)	0.666 (0.653 – 0.679)	0.329 (0.324 – 0.334)	0.265 (0.240 – 0.300)	0.969 (0.968 – 0.971)	0.954 (0.952 – 0.956)
BERT	Bio_ClinicalBERT	0	0.279 (0.276 – 0.283)	0.689 (0.676 – 0.699)	0.311 (0.307 – 0.315)	0.256 (0.252 – 0.261)	0.966 (0.964 – 0.967)	0.962 (0.959 – 0.964)
BERT	bert-base-uncased	0	0.295 (0.285 – 0.305)	0.691 (0.682 – 0.701)	0.328 (0.322 – 0.335)	0.334 (0.290 – 0.378)	0.969 (0.967 – 0.971)	0.962 (0.959 – 0.964)
BERT	ClinicalBERT	0	0.308 (0.298 – 0.318)	0.684 (0.672 – 0.693)	0.363 (0.356 – 0.371)	0.288 (0.269 – 0.309)	0.971 (0.969 – 0.972)	0.954 (0.952 – 0.957)
LLM	MedLlama3-8B	0	0.403 (0.392 – 0.413)	0.198 (0.190 – 0.209)	0.928 (0.914 – 0.940)	0.304 (0.293 – 0.314)	0.988 (0.985 – 0.992)	0.628 (0.623 – 0.634)
LLM	Llama3-Med42–70B	0	0.608 (0.591 – 0.626)	0.661 (0.646 – 0.672)	0.988 (0.983 – 0.992)	0.468 (0.451 – 0.485)	0.997 (0.996 – 0.998)	0.908 (0.904 – 0.912)
LLM	Llama3-Med42–8B	10	0.624 (0.605 – 0.644)	0.716 (0.705 – 0.727)	0.963 (0.952 – 0.971)	0.502 (0.483 – 0.522)	0.988 (0.987 – 0.989)	0.933 (0.929 – 0.938)
LLM	Llama3-OpenBioLLM-8B	0	0.677 (0.657 – 0.695)	0.699 (0.690 – 0.708)	0.680 (0.659 – 0.700)	0.728 (0.703 – 0.753)	0.947 (0.944 – 0.949)	0.987 (0.986 – 0.988)
LLM	Llama-3.1–8B-Instruct	0	0.706 (0.691 – 0.722)	0.729 (0.720 – 0.740)	0.941 (0.930 – 0.952)	0.618 (0.602 – 0.637)	0.979 (0.977 – 0.981)	0.957 (0.955 – 0.959)
LLM	DeepSeek-R1-Distill-Llama-8B	5	0.716 (0.694 – 0.736)	0.762 (0.752 – 0.770)	0.799 (0.781 – 0.814)	0.670 (0.639 – 0.696)	0.966 (0.963 – 0.968)	0.974 (0.971 – 0.976)
LLM	Llama3-0penBioLLM-70B	0	0.769 (0.747 – 0.789)	0.787 (0.775 – 0.796)	0.987 (0.980 – 0.994)	0.659 (0.630 – 0.688)	**0.999 (0.998 – 0.999)**	0.941 (0.938 – 0.944)
LLM	gpt-3.5-turbo-0125	10	0.787 (0.772 – 0.800)	0.808 (0.800 – 0.818)	0.939 (0.929 – 0.949)	0.704 (0.686 – 0.720)	0.989 (0.988 – 0.990)	0.973 (0.971 – 0.975)
LLM	Llama-3.1–70B-Instruct	0	0.835 (0.823 – 0.849)	0.828 (0.818 – 0.840)	0.978 (0.967 – 0.986)	0.769 (0.751 – 0.787)	0.997 (0.997 – 0.998)	0.972 (0.970 – 0.974)
LLM	Llama-3.3–70B-Instruct	10	0.852 (0.828 – 0.872)	0.804 (0.776 – 0.826)	**0.992 (0.984 – 0.998)**	0.793 (0.758 – 0.819)	0.996 (0.993 – 0.998)	0.966 (0.960 – 0.971)
LLM	DeepSeek-R1-Distill-Llama-70B	0	0.871 (0.857 – 0.883)	0.860 (0.853 – 0.869)	0.958 (0.948 – 0.970)	0.831 (0.814 – 0.847)	0.991 (0.990 – 0.992)	0.984 (0.983 – 0.985)
LLM	o3-mini-2025-01-31	5	0.883 (0.873 – 0.892)	0.911 (0.904 – 0.917)	0.899 (0.879 – 0.914)	0.893 (0.881 – 0.902)	0.986 (0.985 – 0.988)	**0.994 (0.993 – 0.995)**
LLM	gpt-4o-2024-08-06	0	0.885 (0.874 – 0.897)	0.898 (0.891 – 0.908)	0.961 (0.950 – 0.970)	0.857 (0.837 – 0.875)	0.991 (0.991 – 0.992)	0.989 (0.988 – 0.990)
LLM	Llama-DrugDetector-8B	3	0.909 (0.894 – 0.922)	**0.939 (0.934 – 0.944)**	0.909 (0.891 – 0.924)	**0.914 (0.900 – 0.928)**	0.992 (0.991 – 0.993)	**0.994 (0.993 – 0.995)**
LLM	Llama-DrugDetector-70B	0	**0.917 (0.905 – 0.928)**	0.926 (0.920 – 0.932)	0.959 (0.947 – 0.968)	0.894 (0.882 – 0.907)	0.994 (0.993 – 0.995)	0.993 (0.992 – 0.994)

**Table 6:** Mean F1-Scores on the held-out test set (n=6443) with bootstrapped lower and upper bounds for each detector and drug class. Abbreviations: Methamphetamine (Meth.), Benzodiazepine (Benzo.), Prescription Opioid Misuse (Rx. Opioids), Injection Drug Use (IDU). Overall performance, requiring simultaneous correct classification across all classes, is also reported.

Detector	Shots	Heroin	Cocaine	Meth.	Benzos.	Rx. Opioids	Cannabis	IDU	Any	Overall
biobert-vl.1	0	0.771 (0.758 – 0.787)	0.480 (0.476 – 0.484)	0.497 (0.496 – 0.498)	0.490 (0.489 – 0.491)	0.495 (0.494 – 0.496)	0.502 (0.494 – 0.513)	0.763 (0.752 – 0.773)	0.946 (0.941 – 0.951)	0.276 (0.271 – 0.281)
Bio_ClinicalBERT	0	0.801 (0.790 – 0.815)	0.478 (0.476 – 0.480)	0.497 (0.496 – 0.498)	0.490 (0.488 – 0.491)	0.495 (0.494 – 0.495)	0.495 (0.494 – 0.495)	0.775 (0.763 – 0.788)	0.939 (0.934 – 0.944)	0.279 (0.276 – 0.283)
bert-base-uncased	0	0.822 (0.809 – 0.837)	0.482 (0.477 – 0.488)	0.497 (0.497 – 0.498)	0.490 (0.489 – 0.491)	0.518 (0.495 – 0.549)	0.510 (0.495 – 0.535)	0.765 (0.753 – 0.776)	0.952 (0.946 – 0.958)	0.295 (0.285 – 0.305)
ClinicalBERT	0	0.844 (0.833 – 0.855)	0.477 (0.475 – 0.479)	0.496 (0.495 – 0.497)	0.494 (0.489 – 0.503)	0.495 (0.494 – 0.495)	0.573 (0.543 – 0.604)	0.755 (0.745 – 0.767)	0.944 (0.939 – 0.949)	0.308 (0.298 – 0.318)
MedLlama3-8B	0	0.167 (0.159 – 0.177)	0.680 (0.665 – 0.693)	0.502 (0.492 – 0.513)	0.899 (0.879 – 0.919)	0.658 (0.629 – 0.682)	0.453 (0.444 – 0.462)	0.642 (0.631 – 0.654)	0.414 (0.403 – 0.424)	0.403 (0.392 – 0.413)
Llama3-Med42–70B	0	0.767 (0.755 – 0.781)	0.869 (0.857 – 0.881)	0.732 (0.693 – 0.767)	0.834 (0.814 – 0.854)	0.613 (0.588 – 0.633)	0.725 (0.694 – 0.748)	0.759 (0.747 – 0.768)	0.932 (0.927 – 0.937)	0.608 (0.591 – 0.626)
Llama3-Med42–8B	10	0.731 (0.716 – 0.746)	0.879 (0.869 – 0.891)	0.702 (0.667 – 0.730)	0.802 (0.783 – 0.824)	0.620 (0.598 – 0.642)	0.762 (0.729 – 0.793)	0.894 (0.886 – 0.905)	0.937 (0.931 – 0.943)	0.624 (0.605 – 0.644)
Llama3-OpenBioLLM-8B	0	0.907 (0.898 – 0.918)	0.955 (0.945 – 0.965)	0.780 (0.742 – 0.817)	0.793 (0.758 – 0.823)	0.648 (0.621 – 0.690)	0.852 (0.821 – 0.889)	0.868 (0.858 – 0.879)	0.760 (0.748 – 0.769)	0.677 (0.657 – 0.695)
Llama-3.1–8B-Instruct	0	0.874 (0.861 – 0.884)	0.959 (0.953 – 0.966)	0.739 (0.700 – 0.775)	0.843 (0.826 – 0.866)	0.575 (0.557 – 0.592)	0.904 (0.881 – 0.927)	0.890 (0.880 – 0.899)	0.907 (0.900 – 0.915)	0.706 (0.691 – 0.722)
DeepSeek-R1-Distill-Llama-8B	5	0.880 (0.868 – 0.893)	0.935 (0.923 – 0.944)	0.806 (0.765 – 0.848)	0.817 (0.790 – 0.840)	0.705 (0.666 – 0.737)	0.859 (0.832 – 0.885)	0.883 (0.875 – 0.893)	0.853 (0.843 – 0.861)	0.716 (0.694 – 0.736)
Llama3-0penBioLLM-70B	0	0.831 (0.819 – 0.843)	0.979 (0.973 – 0.986)	0.849 (0.805 – 0.881)	0.891 (0.871 – 0.908)	0.662 (0.637 – 0.688)	0.919 (0.893 – 0.944)	0.929 (0.919 – 0.936)	0.887 (0.880 – 0.894)	0.769 (0.747 – 0.789)
gpt-3.5-turbo-0125	10	0.880 (0.868 – 0.893)	0.973 (0.966 – 0.979)	0.806 (0.769 – 0.843)	0.876 (0.858 – 0.897)	0.714 (0.688 – 0.740)	0.950 (0.928 – 0.973)	0.926 (0.917 – 0.933)	0.949 (0.944 – 0.954)	0.787 (0.772 – 0.800)
Llama-3.1–70B-Instruct	0	0.895 (0.884 – 0.905)	0.992 (0.988 – 0.995)	0.932 (0.902 – 0.955)	0.928 (0.915 – 0.941)	0.655 (0.636 – 0.680)	0.973 (0.959 – 0.988)	0.928 (0.920 – 0.936)	0.976 (0.973 – 0.980)	0.835 (0.823 – 0.849)
Llama-3.3–70B-Instruct	10	0.858 (0.827 – 0.881)	0.983 (0.968 – 0.994)	**1.000 (1.000 – 1.000)**	0.969 (0.941 – 0.993)	0.655 (0.598 – 0.708)	0.975 (0.928 – 1.000)	0.925 (0.906 – 0.946)	0.965 (0.953 – 0.976)	0.852 (0.828 – 0.872)
DeepSeek-R1-Distill-Llama-70B	0	0.891 (0.880 – 0.902)	0.994 (0.990 – 0.996)	0.943 (0.910 – 0.973)	0.952 (0.938 – 0.964)	0.729 (0.699 – 0.759)	0.989 (0.979 – 0.998)	0.976 (0.971 – 0.981)	0.961 (0.956 – 0.967)	0.871 (0.857 – 0.883)
o3-mini-2025-01-31	5	0.985 (0.980 – 0.990)	0.993 (0.990 – 0.996)	0.975 (0.961 – 0.989)	0.888 (0.864 – 0.910)	0.726 (0.697 – 0.753)	0.983 (0.966 – 0.994)	**0.993 (0.990 – 0.996)**	0.948 (0.942 – 0.954)	0.883 (0.873 – 0.892)
gpt-4o-2024-08-06	0	0.965 (0.958 – 0.971)	0.988 (0.983 – 0.993)	0.962 (0.935 – 0.981)	**0.971 (0.961 – 0.980)**	0.690 (0.662 – 0.714)	0.975 (0.959 – 0.988)	0.987 (0.983 – 0.991)	0.965 (0.961 – 0.969)	0.885 (0.874 – 0.897)
Llama-DrugDetector-8B	3	0.981 (0.977 – 0.986)	0.991 (0.986 – 0.995)	0.948 (0.919 – 0.972)	0.932 (0.912 – 0.948)	**0.830 (0.791 – 0.868)**	0.977 (0.961 – 0.989)	0.983 (0.979 – 0.987)	0.966 (0.961 – 0.970)	0.909 (0.894 – 0.922)
Llama-DrugDetector-70B	0	0.943 (0.935 – 0.951)	**0.994 (0.991 – 0.997)**	0.964 (0.940 – 0.982)	0.969 (0.957 – 0.981)	0.815 (0.784 – 0.848)	**0.989 (0.981 – 0.996)**	0.990 (0.987 – 0.993)	**0.976 (0.973 – 0.981)**	**0.917 (0.905 – 0.928)**

**Table 7:** Summary of common errors made by the best performing detector – our fine-tuned DrugDetector-70B. Percentages are calculated relative to the full test set and may slightly overstate error rates, as multiple issues can occur within a single instance.

Error Type	Error Description	Count	Percent
Insufficient Evidence	Assuming IDVU implied heroin when never explicitly mentioned	126	1.96%
	Assuming illicit use of prescription opioids without evidence	103	1.60%
	Assuming illicit use of benzodiazepines without evidence	9	0.14%
	Assuming specific substance mentioned in vague sentence	8	0.12%
	Assuming opiate/opioid use means heroin use	7	0.11%
	Assuming HepC means IVDU when never explicitly mentioned	5	0.08%
Missed Evidence	Simple failures to identify substance	113	1.75%
	Missed negation of drug use (e.g. patient denied use)	11	0.17%
	Missed illicit nature of use (e.g. prescription opioids)	2	0.03%
Confusion	Hallucinated drug use where none existed	30	0.47%
	Confusing family substance use for patient use	4	0.06%
	Confusing medical recommendation for actual use	3	0.05%
	Confusing typos (e.g. Klopopins → Klonopin)	2	0.03%
	**Total**	**433**	**6.72%**

**Figure A.2:**
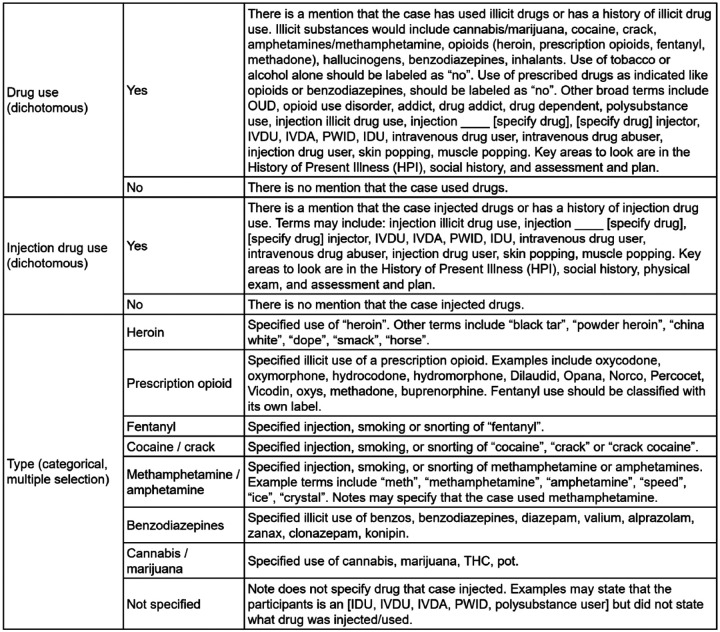
Substance Annotation Guidelines

## Data Availability

The article’s data are from the National Institutes of Health-funded project to predict fatal and non-fatal overdoses (grant number: 1R01DA057630–01). The data cannot be shared publicly due to MIMIC licensing. Investigators who would like to use the data can make specific requests to collaborate by contacting the authors and demonstrating completion of the required training for *CITI Data or Specimens Only Research*.

## Footnotes

2 To further control for class imbalance, we normalize precision, recall, and F1 with *macro-averaging*. This calculates metrics for each label via their *unweighted* means, effectively ignoring overall support for each class. This results in *lowering scores* relative to alternative normalizations like micro- or weighted-averaging, which yield substantially higher scores due to stronger performance of negative classes. For more details, see: https://scikit-learn.org/stable/modules/generated/sklearn.metrics.f1_score.html

3 We fine-tune DeepSeek-R1-Distill-Llama (8B and 70B) based on the best performance observed across both zero- and few-shot testing. The performance of DeepSeek-R1-Distill-Llama-8B was significantly improved in the few-shot setting (F1: 0.716 at 5-shot vs F1: 0.706 for Llama-3.1–8B-Instruct’s best.)

4 Recall that “Overall” reflects the correct identification of all substances present in the medical text simultaneously – i.e., a single misclassified drug nullifies otherwise correct multi-label predictions. This explains the divergence between peak single-substance F1 (0.994) and overall performance (0.917) for Llama-DrugDetector-70B.

5
https://huggingface.co/fabriceyhc/Llama-DrugDetector-70B

## Appendix A. Annotation Guide

We provide detailed guidelines and clarifications for annotators involved in the study. The section begins with the Substance Annotation Guide in A.2, which outlines the specific instructions for categorizing and recording mentions of substance use in patient chart notes. Following the table, a series of clarifications address common scenarios and provide precise rules for annotators to follow. This comprehensive guide ensures consistency and accuracy across all annotations.

### Annotator clarifications:

1. Any mention of injection drug use (IDU or IVDU) will be marked as such and also as drug use – yes
2. Annotations must be within a single sentence
3. Annotate for every substance that is mentioned (if multiple are mentioned)
4. If there is a drug name mentioned, check to see which drug class it falls under, and annotate with the respected drug class.
5. “not specified” will account for notes that mention: “polysubstance abuse”, “substance abuse”, “using drugs”, mentions “opiates” broadly, etc.
  - Disclaimer: IVDU is NOT marked as “not specified”
6. Chart notes that indicate patient denies or does not take drugs will be marked as drug use no, and the corresponding substances mentioned will not be annotated.
  - Ex: ‘patient denies using heroin’
    - Annotation: Drug use no. No mark for heroin.
  - Ex: ‘does not use heroin or cocaine’
    - Annotation: Drug use no. No mark for heroin or cocaine.
7. If the chart mentioned “no IDU”, there will also be an annotation for “drug use no”
8. Make sure all drug use that is being annotated is illicit drug use, and not prescribed medication.
9. For unclear opioid examples in which the medical note mentions “opiate overdose” or “opioid withdrawal”, mark as “not specified”
  - Only mark as prescription opioid if it explicitly says so

## Appendix B. Drug Co-occurences

**Table B.8:** Co-occurence of substances in the test-split of the DrugDetection dataset. Abbreviations: Methamphetamine (Meth.), Benzodiazepine (Benzo.), Prescription Opioid Misuse (Rx. Opioids), Injection Drug Use (IDU).

Drugs	Heroin	Cocaine	Meth.	Benzo.	Rx. Opioids	Cannabis	IDU
**Heroin**	749	196	18	43	26	32	254
**Cocaine**	196	528	20	56	11	44	120
**Meth.**	18	20	72	11	1	3	21
**Benzo.**	43	56	11	232	28	13	21
**Rx. Opioids**	26	11	1	28	122	5	22
**Cannabis**	32	44	3	13	5	121	14
**IDU**	254	120	21	21	22	14	1041

Table B.8 presents the co-occurrence matrix of various substances within the test split of the DrugDetection dataset, shedding light on the relationships between different drugs. The diagonal entries indicate the frequency of individuals using each specific substance, with injecting drug use (IDU) being the most common at 1041 occurrences. Significant co-occurrences are observed between heroin and cocaine (196 cases) and between heroin and IDU (254 cases), reflecting a notable overlap among these substances. This table highlights the prevalence of polysubstance use within the dataset, particularly with regard to IDU, heroin, and cocaine.

## Appendix C. Model Descriptions

We investigate a range of language models, categorized into two main classes: BERT-style encoders and GPT-style decoders. These models are further explored across different dimensions, including base models, models with generic medical pre-training, and models with fine-tuning.

### BERT-Style Encoders

BERT-style encoders are designed to convert input text into rich, contextualized representations. They excel in tasks that require a deep understanding of the text, such as text classification. We selected models from this category due to their proven effectiveness in capturing the meaning and relationships within medical and clinical texts.

1. **BERT** [17]: The standard BERT model, trained on a large corpus of general English text, serving as a baseline for comparison. Model source: https://huggingface.co/google-bert/bert-base-uncased.
2. **BioBERT** [18]: A variant of BERT pre-trained on Pubmed datasets [55] containing 18 billion words from biomedical literature and has demonstrated an improved ability to process biomedical text. Model source: https://huggingface.co/dmis-lab/biobert-v1.1.
3. **ClinicalBERT** [20]: A variant of BERT pre-trained pre-trained on clinical text (MIMIC III) [30], providing specialized adaptation to clinical language. Model source: https://huggingface.co/medicalai/ClinicalBERT.
4. **Bio_ClinicalBERT** [19]: An extension of BioBERT with additional training on clinical notes from MIMIC III, enhancing its ability to handle clinical language and terminologies. Model source: https://huggingface.co/emilyalsentzer/Bio_ClinicalBERT

### GPT-Style Decoders

In contrast, GPT-style decoders are designed for text generation, predicting the next word in a sequence based on the preceding context. We adapt these decoders for classification tasks by prompting them to generate responses that mimic classification outputs. We study a mix of proprietary LLMs – GPT-3.5-Turbo [21], GPT-4-Turbo [22], and GPT-4o [36] – as well as open-source alternatives. Among the open-source LLMs, we examine DeepSeek-R1-Distill-Llama [37] and the Llama-3 series of models [23], including Llama-3-Instruct and its updated variant Llama-3.1-Instruct, which offers strong performance across diverse tasks. The open-source LLMs offer comparable performance to most proprietary LLMs and have the distinct advantage of being hosted locally, which is ideal for medical applications where privacy requirements complicate sharing medical data with third-party AI vendors.

1. **gpt-3.5-turbo-0125** [21]: An advanced generative model known for its robust language understanding and generation capabilities.
2. **gpt-4o** [36]: A multimodal variant of GPT-4 [22], optimized for specific applications, providing enhanced capabilities in understanding and generating complex texts.
3. **o3-mini** [56]: A reasoning model optimized for STEM tasks like math, coding, and science, offering adjustable reasoning levels and faster response times compared to its predecessors.
4. **Meta-Llama-3-Instruct** [23]: A series of instruction-following models with different parameter sizes (8B, 70B) designed for general purposes, providing robust performance across various tasks.
  - https://huggingface.co/meta-llama/Meta-Llama-3-8B-Instruct
  - https://huggingface.co/meta-llama/Meta-Llama-3-70B-Instruct
5. **Meta-Llama-3.1-Instruct** [23]: An updated series of the Meta-Llama-3 models supporting longer inputs, improved multilingualism, and other improvements in three parameter sizes (8B, 70B, 405B).
  - https://huggingface.co/meta-llama/Meta-Llama-3.1-8B-Instruct
  - https://huggingface.co/meta-llama/Meta-Llama-3.1-70B-Instruct
  - https://huggingface.co/meta-llama/Meta-Llama-3.1-405B-Instruct (Not included in study)
6. **Meta-Llama-3.3-Instruct** [23]: A more efficient and powerful version of the 70B model of the Llama-3 family, generally regarded as being on par with Meta-Llama-3.1–405B-Instruct at a fraction of the size.
  - https://huggingface.co/meta-llama/Llama-3.3-70B-Instruct
7. **DeepSeek-R1-Distill-Llama** [37]: A fine-tuned version of Llama-3.1 specifically optimized for mathematical, scientific, and logical reasoning capabilities.
  - https://huggingface.co/deepseek-ai/DeepSeek-R1-Distill-Llama-8B
  - https://huggingface.co/deepseek-ai/DeepSeek-R1-Distill-Llama-70B
8. **MedLlama3** [38]: The model is a fine-tuned version of Llama-3–8B using publicly available medical data.
  - https://huggingface.co/ProbeMedicalYonseiMAILab/medllama3-v20^6^
9. **Llama3-Med42** [40]: A collection of open-access clinical LLMs developed by M42, designed to enhance accessibility to medical knowledge. Based on Llama-3 and available with either 8 or 70 billion parameters, these generative AI systems deliver high-quality responses to medical questions.
  - https://huggingface.co/m42-health/Llama3-Med42-8B
  - https://huggingface.co/m42-health/Llama3-Med42-70B
10. **Llama3-OpenBioLLM** [39]: Similar to Llama3-Med42, these models are developed by Saama AI Labs and pre-trained on a diverse set of biomedical literature to support a wide range of biomedical NLP applications.
  - https://huggingface.co/aaditya/Llama3-OpenBioLLM-8B
  - https://huggingface.co/aaditya/Llama3-OpenBioLLM-70B
11. **Llama-DrugDetector**: Our few-shot fine-tuned version of DeepSeek-R1-Distill-Llama optimized for drug detection tasks.
  - https://huggingface.co/fabriceyhc/Llama-DrugDetector-8B
  - https://huggingface.co/fabriceyhc/Llama-DrugDetector-70B

## Appendix D. Training

To better understand the degree to which “off-the-shelf” models can be improved with few-shot training, we employed a comprehensive fine-tuning process on both BERT-style models and GPT-style LLMs.

We fine-tuned all four BERT-style models – BERT, BioBERT, ClinicalBERT, and Bio_ClinicalBERT – using our DrugDetection dataset. Since these models are relatively small (less than 1B parameters), they train in less than 2 hours each on a single Nvidia A6000 48GB GPU. For the fine-tuning process, we utilized SetFit [57] with a multi-output target strategy to facilitate multi-label training. The input to the models consisted solely of the medical note, while the output is an 8-dimensional binary vector, indicating the presence or absence of each drug class within the note. The models were trained for up to 20 epochs, with an early stopping criterion if the validation loss did not decrease after 10 attempts. On average, the models achieved convergence within 1.65 epochs.

Given the resource-intensive and time-consuming nature of training LLMs, we limited our focus to the Deepseek-R1-Distill-Llama models with 8 billion (8B) and 70 billion (70B) parameters. We used llama-factory [58] scripts for supervised fine-tuning with Low-Rank Adaptation (LoRA) [59] to train these models on the DrugDetection dataset. The training process for both models spanned 3 epochs, without early stopping, as this feature is not currently supported in the utilized framework. Fine-tuning the 8B model required approximately 8 hours on 2 Nvidia A6000 48GB GPUs, while the 70B required approximately 24hrs on 8 GPUs of the same type.

Standard configurations for supervised fine-tuning were applied, including using an AdamW optimizer [60] with a weight decay of 0.01 and a learning rate of 0.0003. Additionally, a 100-step cosine warmup schedule was implemented. Cross-entropy loss was computed between the expected labels and the generated outputs to guide the optimization process.

## Appendix E. Prompt Template

After extensive iterations, we developed an effective prompt template. Our approach involved prototyping a template, testing it on a small development set (separate from the DrugDetection dataset), and analyzing the errors made by the LLMs. These error analyses provided actionable insights, enabling us to add “guard rails” in the special notes section. These adjustments significantly enhanced performance for certain classes, especially for detecting prescription opioid misuse (i.e. initially around ~60% F1 accuracy, up to ~95%). Depending on the LLM, we would either use guidance [42] or langchain [43] with JSON output parsing to prompt for True or False labels for each drug in an extractable format.

Listing 1: Zero-shot drug detection prompt featuring task and drug descriptions with special notes curated from several rounds of error analysis.

You are a medical expert medical expert conducting important research where accuracy is essential.

Task: Analyze the provided medical text for references to illicit drug use, focusing on specific drug categories and adhering to special considerations.

Drug Categories to Identify:

- Heroin: Heroin is an illegal opioid drug known for its high potential for addiction and overdose.
- Cocaine: Cocaine is a powerful stimulant drug that is often abused for its euphoric effects.
- Methamphetamine: Methamphetamine (including illicit amphetamine use, but not prescribed amphetamines for ADHD) is a potent central nervous system stimulant that is highly addictive.
- Benzodiazepine: Benzodiazepines (only if being misused or used illicitly, not if taken as prescribed) are a class of psychoactive drugs commonly prescribed for anxiety, insomnia, and other conditions but can be abused for their sedative effects.
- Prescription Opioids: Prescription opioids (only if being misused or used illicitly, not if taken as prescribed) are medications typically prescribed for pain relief but can be highly addictive when misused.
- Cannabis: Cannabis, also known as marijuana, is often used recreationally or medicinally but can be illegal depending on the jurisdiction.
- Injection Drugs: Injection drug use (IDU, IVDA, IVDU) refers to the use of illicit drugs administered via needles, often associated with higher risks of infectious diseases.
- General Drugs: General drug use refers to the use of any illegal or illicit substances.

Special Considerations:

1. For unspecified ”substance dependence,” mark only ”General Drug Use” as True
2. If no substances or drug–related behaviors are mentioned, mark all categories as False
3. Mark as False if information is missing or not explicitly stated
4. Warnings against drug use do not indicate patient use
5. Medical recommendations about drugs do not indicate patient use
6. Family drug use is not relevant to the patient
7. Only identify inappropriate use of opioids and benzodiazepines (e.g., acquired from friends or streets)
8. Patient denial of drug use should be marked as False

### The medical text to evaluate:

{medical_text}

## Appendix F. Detailed Model Performance By Drug Class

We provide detailed breakdowns of detector performance by drug class, with each table focusing on a specific metric. For instance, Table F.9 shows accuracy. Detectors are sorted by “Overall” performance, which reflects exact matches across all classes simultaneously. This “Overall” metric is often lower due to the complexity added by the frequent co-occurrence of multiple drug classes in medical notes.

**Table F.9:** Mean accuracy on the held-out test set (n=6443) with bootstrapped confidence intervals for each detector and drug class. The “Overall” scores may be lower than those for individual classes, as they reflect exact matches across all classes simultaneously, a task made more challenging by the frequent co-occurrence of multiple drug classes in many medical notes. Abbreviations: Methamphetamine (Meth.), Benzodiazepine (Benzo.), Prescription Opioid Misuse (Rx. Opioids), Injection Drug Use (IDU).

Detector	Shots	Heroin	Cocaine	Meth.	Benzo.	Rx. Opioids	Cannabis	IDU	Any	Overall
MedLlama3-v20-8B	3	0.344 (0.335 – 0.356)	0.845 (0.834 – 0.855)	0.916 (0.909 – 0.922)	0.922 (0.916 – 0.928)	0.818 (0.809 – 0.826)	0.845 (0.837 – 0.853)	0.475 (0.462 – 0.489)	0.589 (0.578 – 0.601)	0.198 (0.190 – 0.209)
Llama3-Med42–70B	0	0.859 (0.850 – 0.867)	0.950 (0.946 – 0.955)	0.976 (0.972 – 0.979)	0.967 (0.963 – 0.971)	0.905 (0.899 – 0.912)	0.958 (0.954 – 0.962)	0.820 (0.810 – 0.828)	0.932 (0.926 – 0.938)	0.661 (0.646 – 0.672)
biobert-vl.1	0	0.893 (0.886 – 0.901)	0.915 (0.908 – 0.922)	0.988 (0.985 – 0.990)	0.961 (0.957 – 0.966)	0.980 (0.977 – 0.983)	0.980 (0.977 – 0.983)	0.827 (0.818 – 0.835)	0.946 (0.940 – 0.950)	0.666 (0.653 – 0.679)
ClinicalBERT	0	0.925 (0.919 – 0.931)	0.913 (0.906 – 0.919)	0.985 (0.982 – 0.987)	0.962 (0.958 – 0.967)	0.979 (0.976 – 0.982)	0.975 (0.972 – 0.979)	0.826 (0.818 – 0.832)	0.944 (0.940 – 0.949)	0.684 (0.672 – 0.693)
Bio_ClinicalBERT	0	0.914 (0.906 – 0.921)	0.915 (0.909 – 0.922)	0.988 (0.985 – 0.991)	0.959 (0.955 – 0.964)	0.979 (0.975 – 0.982)	0.978 (0.974 – 0.982)	0.853 (0.844 – 0.862)	0.939 (0.932 – 0.944)	0.689 (0.676 – 0.699)
bert-base-uncased	0	0.929 (0.923 – 0.934)	0.917 (0.912 – 0.922)	0.989 (0.986 – 0.991)	0.961 (0.957 – 0.965)	0.979 (0.975 – 0.982)	0.980 (0.977 – 0.983)	0.830 (0.820 – 0.839)	0.952 (0.947 – 0.958)	0.691 (0.682 – 0.701)
Llama3-OpenBioLLM-8B	0	0.962 (0.958 – 0.966)	0.987 (0.984 – 0.989)	0.985 (0.982 – 0.987)	0.977 (0.974 – 0.980)	0.964 (0.960 – 0.968)	0.989 (0.986 – 0.991)	0.931 (0.925 – 0.936)	0.774 (0.764 – 0.783)	0.699 (0.690 – 0.708)
Llama3-Med42–8B	10	0.827 (0.816 – 0.838)	0.955 (0.950 – 0.960)	0.969 (0.964 – 0.975)	0.959 (0.953 – 0.964)	0.911 (0.904 – 0.917)	0.970 (0.965 – 0.975)	0.934 (0.928 – 0.939)	0.938 (0.932 – 0.942)	0.716 (0.705 – 0.727)
Llama-3.1–8B-Instruct	10	0.883 (0.875 – 0.891)	0.978 (0.975 – 0.981)	0.985 (0.982 – 0.988)	0.978 (0.975 – 0.982)	0.885 (0.877 – 0.892)	0.989 (0.986 – 0.991)	0.945 (0.939 – 0.950)	0.950 (0.947 – 0.955)	0.729 (0.720 – 0.740)
DeepSeek-R1-Distill-Llama-8B	10	0.919 (0.912 – 0.925)	0.978 (0.975 – 0.981)	0.985 (0.982 – 0.987)	0.977 (0.974 – 0.981)	0.968 (0.965 – 0.971)	0.987 (0.985 – 0.989)	0.938 (0.932 – 0.943)	0.892 (0.884 – 0.898)	0.762 (0.752 – 0.770)
Llama3-0penBioLLM-70B	10	0.885 (0.877 – 0.894)	0.980 (0.977 – 0.983)	0.983 (0.981 – 0.986)	0.972 (0.968 – 0.976)	0.910 (0.903 – 0.917)	0.985 (0.982 – 0.988)	0.963 (0.959 – 0.968)	0.968 (0.964 – 0.972)	0.787 (0.775 – 0.796)
Llama-3.3-70B-Instruct	10	0.923 (0.906 – 0.941)	0.994 (0.989 – 0.999)	**1.000 (1.000 – 1.000)**	0.994 (0.988 – 0.999)	0.923 (0.904 – 0.940)	0.999 (0.996 – 1.000)	0.955 (0.944 – 0.967)	0.966 (0.953 – 0.977)	0.804 (0.776 – 0.826)
gpt-3.5-turbo-0125	10	0.942 (0.936 – 0.947)	0.991 (0.989 – 0.993)	0.987 (0.984 – 0.990)	0.978 (0.974 – 0.982)	0.962 (0.957 – 0.966)	0.996 (0.994 – 0.998)	0.957 (0.952 – 0.963)	0.948 (0.943 – 0.953)	0.808 (0.800 – 0.818)
Llama-3.1–70B-Instruct	0	0.949 (0.944 – 0.955)	0.997 (0.996 – 0.999)	0.997 (0.996 – 0.998)	0.989 (0.986 – 0.991)	0.930 (0.924 – 0.936)	0.998 (0.997 – 0.999)	0.957 (0.952 – 0.962)	0.976 (0.973 – 0.979)	0.828 (0.818 – 0.840)
DeepSeek-R1-Distill-Llama-70B	10	0.936 (0.931 – 0.942)	0.994 (0.992 – 0.996)	0.995 (0.993 – 0.997)	0.990 (0.988 – 0.992)	0.970 (0.966 – 0.975)	0.998 (0.996 – 0.999)	0.989 (0.986 – 0.991)	0.957 (0.953 – 0.961)	0.860 (0.853 – 0.869)
gpt-4o-2024-08-06	0	0.985 (0.982 – 0.988)	0.996 (0.995 – 0.998)	0.998 (0.997 – 0.999)	0.996 (0.994 – 0.997)	0.949 (0.943 – 0.955)	0.998 (0.997 – 0.999)	0.993 (0.990 – 0.994)	0.965 (0.960 – 0.969)	0.898 (0.891 – 0.908)
o3-mini-2025-01-31	10	0.994 (0.991 – 0.995)	0.998 (0.997 – 0.999)	0.999 (0.998 – 1.000)	0.987 (0.984 – 0.990)	0.968 (0.964 – 0.971)	0.999 (0.998 – 1.000)	**0.996 (0.994 – 0.997)**	0.953 (0.948 – 0.958)	0.911 (0.904 – 0.917)
Llama-DrugDetector-70B	0	0.975 (0.972 – 0.978)	0.998 (0.997 – 0.999)	0.998 (0.997 – 0.999)	**0.996 (0.994 – 0.997)**	0.980 (0.977 – 0.984)	**0.999 (0.999 – 1.000)**	0.995 (0.994 – 0.997)	**0.976 (0.973 – 0.980)**	0.926 (0.920 – 0.932)
Llama-DrugDetector-8B	0	**0.996 (0.994 – 0.997)**	**0.998 (0.997 – 0.999)**	0.997 (0.996 – 0.998)	0.989 (0.987 – 0.991)	**0.986 (0.983 – 0.989)**	0.998 (0.998 – 0.999)	0.993 (0.991 – 0.995)	0.975 (0.971 – 0.978)	**0.939 (0.934 – 0.944)**

**Table F.10:** Mean Sensitivity (recall) on the held-out test set (n=6443) with bootstrapped lower and upper bounds for each detector and drug class. Abbreviations: Methamphetamine (Meth.), Benzodiazepine (Benzo.), Prescription Opioid Misuse (Rx. Opioids), Injection Drug Use (IDU).

Detector	Shots	Heroin	Cocaine	Meth.	Benzos.	Rx. Opioids	Cannabis	IDU	Any	Overall
Bio_Clini calBERT	0	0.820 (0.807 – 0.832)	0.498 (0.498 – 0.499)	0.500 (0.499 – 0.500)	0.497 (0.497 – 0.498)	0.499 (0.498 – 0.499)	0.499 (0.498 – 0.499)	0.838 (0.826 – 0.850)	0.939 (0.934 – 0.945)	0.311 (0.307 – 0.315)
bert-base-uncased	0	0.817 (0.803 – 0.833)	0.501 (0.499 – 0.504)	0.500 (0.500 – 0.500)	0.499 (0.498 – 0.499)	0.512 (0.499 – 0.530)	0.507 (0.499 – 0.521)	0.872 (0.864 – 0.879)	0.952 (0.947 – 0.958)	0.328 (0.322 – 0.335)
biobert-v1.1	0	0.809 (0.795 – 0.824)	0.499 (0.498 – 0.502)	0.500 (0.499 – 0.500)	0.499 (0.498 – 0.499)	0.500 (0.499 – 0.500)	0.503 (0.499 – 0.509)	0.875 (0.867 – 0.882)	0.946 (0.942 – 0.951)	0.329 (0.324 – 0.334)
ClinicalBERT	0	0.913 (0.901 – 0.923)	0.497 (0.496 – 0.498)	0.498 (0.497 – 0.499)	0.501 (0.499 – 0.506)	0.499 (0.498 – 0.499)	0.558 (0.534 – 0.586)	0.858 (0.848 – 0.867)	0.944 (0.939 – 0.949)	0.363 (0.356 – 0.371)
Llama3-0penBioLLM-8B	0	0.903 (0.891 – 0.915)	0.943 (0.929 – 0.957)	0.921 (0.878 – 0.960)	0.732 (0.696 – 0.761)	0.700 (0.660 – 0.744)	0.850 (0.815 – 0.888)	0.851 (0.840 – 0.863)	0.769 (0.759 – 0.776)	0.680 (0.659 – 0.700)
DeepSeek-R1-Distill-Llama-8B	10	0.940 (0.933 – 0.948)	0.964 (0.953 – 0.972)	0.947 (0.923 – 0.974)	0.859 (0.827 – 0.886)	0.784 (0.737 – 0.828)	0.907 (0.877 – 0.935)	0.899 (0.889 – 0.908)	0.890 (0.881 – 0.899)	0.829 (0.810 – 0.845)
o3-mini-2025-01-31	0	**0.989 (0.984 – 0.993)**	0.993 (0.987 – 0.997)	0.969 (0.937 – 0.993)	0.834 (0.801 – 0.855)	0.876 (0.836 – 0.913)	0.979 (0.962 – 0.993)	0.992 (0.988 – 0.996)	0.942 (0.936 – 0.947)	0.899 (0.884 – 0.911)
Llama-DrugDetector-8B	3	**0.989 (0.984 – 0.993)**	0.991 (0.984 – 0.996)	0.958 (0.925 – 0.986)	0.960 (0.944 – 0.978)	0.795 (0.752 – 0.836)	0.975 (0.954 – 0.991)	0.977 (0.971 – 0.983)	0.966 (0.961 – 0.970)	0.909 (0.891 – 0.924)
gpt-3.5-turbo-0125	10	0.948 (0.940 – 0.956)	0.987 (0.981 – 0.993)	0.979 (0.959 – 0.994)	0.968 (0.953 – 0.981)	0.886 (0.853 – 0.913)	0.975 (0.954 – 0.991)	0.956 (0.949 – 0.963)	0.948 (0.943 – 0.954)	0.939 (0.929 – 0.949)
MedLlama3-8B	5	0.626 (0.620 – 0.631)	0.914 (0.909 – 0.920)	0.945 (0.917 – 0.963)	0.935 (0.920 – 0.950)	0.856 (0.822 – 0.882)	0.893 (0.864 – 0.912)	0.669 (0.662 – 0.675)	0.592 (0.586 – 0.598)	0.955 (0.943 – 0.966)
DeepSeek-R1-Distill-Llama-70B	0	0.965 (0.960 – 0.970)	**0.995 (0.990 – 0.998)**	0.941 (0.905 – 0.977)	0.978 (0.962 – 0.989)	0.952 (0.934 – 0.968)	0.991 (0.976 – 1.000)	0.988 (0.984 – 0.991)	0.961 (0.955 – 0.966)	0.958 (0.948 – 0.970)
Llama-DrugDetector-70B	0	0.981 (0.977 – 0.985)	0.993 (0.988 – 0.997)	0.946 (0.913 – 0.974)	0.976 (0.960 – 0.988)	0.954 (0.926 – 0.976)	0.990 (0.977 – 1.000)	**0.993 (0.990 – 0.995)**	**0.976 (0.973 – 0.980)**	0.959 (0.947 – 0.968)
gpt-4o-2024-08-06	3	0.967 (0.960 – 0.973)	0.988 (0.980 – 0.994)	0.993 (0.979 – 0.999)	0.970 (0.956 – 0.982)	0.953 (0.934 – 0.967)	0.983 (0.965 – 0.997)	0.988 (0.984 – 0.991)	0.968 (0.962 – 0.972)	0.967 (0.957 – 0.975)
Llama-3.1–8B-Instruct	3	0.911 (0.903 – 0.917)	0.979 (0.973 – 0.985)	0.979 (0.962 – 0.987)	0.973 (0.963 – 0.982)	0.899 (0.874 – 0.918)	0.979 (0.965 – 0.991)	0.948 (0.943 – 0.953)	0.940 (0.934 – 0.946)	0.967 (0.958 – 0.975)
Llama3-Med42–8B	0	0.906 (0.901 – 0.912)	0.978 (0.974 – 0.980)	0.971 (0.957 – 0.981)	0.966 (0.958 – 0.976)	0.901 (0.887 – 0.914)	0.962 (0.942 – 0.979)	0.962 (0.957 – 0.967)	0.937 (0.932 – 0.943)	0.968 (0.961 – 0.975)
Llama-3.1–70B-Instruct	5	0.933 (0.926 – 0.938)	0.990 (0.985 – 0.995)	0.982 (0.959 – 0.998)	**0.991 (0.986 – 0.994)**	0.938 (0.926 – 0.950)	0.989 (0.976 – 0.998)	0.966 (0.962 – 0.970)	0.971 (0.968 – 0.975)	0.983 (0.975 – 0.989)
Llama3-Med42–70B	10	0.853 (0.847 – 0.860)	0.955 (0.950 – 0.959)	0.979 (0.977 – 0.982)	0.975 (0.969 – 0.979)	0.913 (0.897 – 0.928)	0.968 (0.954 – 0.980)	0.885 (0.880 – 0.892)	0.931 (0.925 – 0.937)	0.990 (0.985 – 0.995)
Llama3-0penBioLLM-70B	5	0.923 (0.917 – 0.928)	0.986 (0.984 – 0.988)	0.989 (0.988 – 0.991)	0.977 (0.968 – 0.984)	0.936 (0.923 – 0.949)	0.982 (0.967 – 0.990)	0.973 (0.970 – 0.976)	0.969 (0.966 – 0.973)	0.991 (0.984 – 0.996)
Llama-3.3–70B-Instruct	10	0.956 (0.948 – 0.964)	0.991 (0.978 – 0.999)	**1.000 (1.000 – 1.000)**	0.987 (0.956 – 0.999)	**0.960 (0.951 – 0.968)**	**0.999 (0.998 – 1.000)**	0.973 (0.967 – 0.981)	0.965 (0.954 – 0.976)	**0.992 (0.984 – 0.998)**

**Table F.11:** Mean Positive Predictive Value (precision) on the held-out test set (n=6443) with bootstrapped lower and upper bounds for each detector and drug class. Abbreviations: Methamphetamine (Meth.), Benzodiazepine (Benzo.), Prescription Opioid Misuse (Rx. Opioids), Injection Drug Use (IDU).

Detector	Shots	Heroin	Cocaine	Meth.	Benzos.	Rx. Opioids	Cannabis	IDU	Any	Overall
Bio_Clini calBERT	0	0.786 (0.772 – 0.803)	0.459 (0.456 – 0.463)	0.494 (0.493 – 0.496)	0.482 (0.480 – 0.484)	0.491 (0.489 – 0.492)	0.491 (0.489 – 0.492)	0.746 (0.733 – 0.758)	0.940 (0.935 – 0.945)	0.256 (0.252 – 0.261)
biobert-v1.1	0	0.745 (0.731 – 0.761)	0.486 (0.456 – 0.551)	0.494 (0.493 – 0.496)	0.482 (0.480 – 0.484)	0.490 (0.489 – 0.492)	0.559 (0.489 – 0.690)	0.735 (0.724 – 0.744)	0.947 (0.943 – 0.952)	0.265 (0.240 – 0.300)
ClinicalBERT	0	0.802 (0.790 – 0.814)	0.459 (0.455 – 0.462)	0.494 (0.493 – 0.496)	0.517 (0.480 – 0.578)	0.490 (0.489 – 0.492)	0.601 (0.558 – 0.653)	0.728 (0.718 – 0.737)	0.944 (0.940 – 0.949)	0.288 (0.269 – 0.309)
MedLlama3-8B	0	0.561 (0.558 – 0.566)	0.653 (0.642 – 0.663)	0.528 (0.523 – 0.534)	0.903 (0.881 – 0.927)	0.610 (0.589 – 0.630)	0.527 (0.523 – 0.531)	0.667 (0.659 – 0.677)	0.719 (0.702 – 0.734)	0.304 (0.293 – 0.314)
bert-base-uncased	0	0.828 (0.810 – 0.843)	0.551 (0.459 – 0.661)	0.494 (0.493 – 0.495)	0.482 (0.480 – 0.484)	0.580 (0.491 – 0.683)	0.586 (0.490 – 0.733)	0.736 (0.725 – 0.746)	0.952 (0.948 – 0.958)	0.334 (0.290 – 0.378)
Llama3-Med42–70B	0	0.725 (0.714 – 0.738)	0.810 (0.797 – 0.825)	0.656 (0.624 – 0.687)	0.762 (0.739 – 0.784)	0.580 (0.565 – 0.593)	0.655 (0.631 – 0.674)	0.734 (0.725 – 0.743)	0.935 (0.930 – 0.940)	0.468 (0.451 – 0.485)
Llama3-Med42–8B	10	0.700 (0.687 – 0.711)	0.824 (0.813 – 0.839)	0.634 (0.607 – 0.656)	0.732 (0.713 – 0.755)	0.585 (0.570 – 0.599)	0.688 (0.659 – 0.717)	0.857 (0.847 – 0.871)	0.940 (0.934 – 0.945)	0.502 (0.483 – 0.522)
Llama-3.1–8B-Instruct	0	0.824 (0.809 – 0.837)	0.932 (0.920 – 0.942)	0.664 (0.631 – 0.697)	0.784 (0.767 – 0.810)	0.561 (0.550 – 0.571)	0.846 (0.813 – 0.877)	0.856 (0.845 – 0.866)	0.921 (0.915 – 0.928)	0.618 (0.602 – 0.637)
Llama3-0penBioLLM-70B	0	0.780 (0.767 – 0.792)	0.963 (0.953 – 0.976)	0.773 (0.725 – 0.815)	0.828 (0.803 – 0.851)	0.610 (0.592 – 0.629)	0.873 (0.835 – 0.913)	0.897 (0.884 – 0.906)	0.904 (0.899 – 0.911)	0.659 (0.630 – 0.688)
DeepSeek-R1-Distill-Llama-8B	5	0.836 (0.821 – 0.851)	0.911 (0.894 – 0.925)	0.737 (0.694 – 0.776)	0.821 (0.789 – 0.852)	0.666 (0.631 – 0.699)	0.822 (0.793 – 0.850)	0.878 (0.866 – 0.890)	0.873 (0.865 – 0.881)	0.670 (0.639 – 0.696)
gpt-3.5-turbo-0125	10	0.836 (0.822 – 0.853)	0.959 (0.950 – 0.967)	0.728 (0.689 – 0.769)	0.816 (0.794 – 0.844)	0.653 (0.630 – 0.676)	0.927 (0.897 – 0.963)	0.902 (0.890 – 0.912)	0.949 (0.945 – 0.955)	0.704 (0.686 – 0.720)
Llama-3.1–70B-Instruct	0	0.848 (0.835 – 0.861)	0.989 (0.985 – 0.994)	0.912 (0.871 – 0.946)	0.882 (0.863 – 0.901)	0.605 (0.592 – 0.624)	0.958 (0.936 – 0.981)	0.895 (0.884 – 0.906)	0.976 (0.973 – 0.980)	0.769 (0.751 – 0.787)
Llama3-0penBioLLM-8B	10	0.872 (0.856 – 0.886)	0.965 (0.956 – 0.973)	0.822 (0.779 – 0.873)	0.898 (0.863 – 0.934)	0.687 (0.659 – 0.723)	0.954 (0.921 – 0.986)	0.884 (0.871 – 0.897)	0.836 (0.827 – 0.843)	0.791 (0.772 – 0.812)
Llama-3.3–70B-Instruct	10	0.808 (0.772 – 0.836)	0.974 (0.954 – 0.993)	**1.000 (1.000 – 1.000)**	0.953 (0.912 – 0.988)	0.607 (0.569 – 0.647)	0.955 (0.875 – 1.000)	0.891 (0.863 – 0.919)	0.965 (0.954 – 0.975)	0.793 (0.758 – 0.819)
DeepSeek-R1-Distill-Llama-70B	0	0.844 (0.832 – 0.858)	0.992 (0.988 – 0.997)	0.947 (0.907 – 0.983)	0.929 (0.906 – 0.950)	0.660 (0.635 – 0.685)	0.987 (0.973 – 0.998)	0.965 (0.958 – 0.972)	0.964 (0.960 – 0.969)	0.831 (0.814 – 0.847)
gpt-4o-2024-08-06	0	0.952 (0.942 – 0.961)	0.993 (0.988 – 0.997)	0.945 (0.904 – 0.980)	0.955 (0.939 – 0.970)	0.630 (0.609 – 0.649)	0.969 (0.941 – 0.989)	0.983 (0.978 – 0.988)	0.967 (0.963 – 0.971)	0.857 (0.837 – 0.875)
o3-mini-2025-01-31	5	0.982 (0.975 – 0.988)	0.996 (0.992 – 0.998)	0.972 (0.946 – 0.992)	**0.969 (0.950 – 0.985)**	0.669 (0.643 – 0.694)	0.984 (0.962 – 0.998)	**0.992 (0.989 – 0.996)**	0.952 (0.947 – 0.958)	0.893 (0.881 – 0.902)
Llama-DrugDetector-70B	0	0.913 (0.902 – 0.924)	**0.996 (0.992 – 0.999)**	0.983 (0.961 – 0.999)	0.962 (0.944 – 0.979)	0.744 (0.713 – 0.781)	**0.989 (0.978 – 1.000)**	0.988 (0.983 – 0.993)	**0.978 (0.974 – 0.982)**	0.894 (0.882 – 0.907)
Llama-DrugDetector-8B	0	**0.988 (0.983 – 0.992)**	0.994 (0.990 – 0.998)	0.950 (0.919 – 0.982)	0.886 (0.862 – 0.909)	**0.939 (0.881 – 0.982)**	0.977 (0.957 – 0.995)	0.988 (0.985 – 0.993)	0.975 (0.971 – 0.979)	**0.929 (0.908 – 0.946)**

**Table F.12:** Mean Negative Predictive Value on the held-out test set (n=6443) with bootstrapped lower and upper bounds for each detector and drug class. Abbreviations: Methamphetamine (Meth.), Benzodiazepine (Benzo.), Prescription Opioid Misuse (Rx. Opioids), Injection Drug Use (IDU).

Detector	Shots	Heroin	Cocaine	Meth.	Benzos.	Rx. Opioids	Cannabis	IDU	Any	Overall
Llama3-0penBioLLM-8B	0	0.977 (0.973 – 0.981)	0.990 (0.987 – 0.993)	0.998 (0.997 – 0.999)	0.980 (0.977 – 0.984)	0.989 (0.986 – 0.991)	0.994 (0.993 – 0.996)	0.950 (0.945 – 0.955)	0.695 (0.681 – 0.710)	0.947 (0.944 – 0.949)
Bio_ClinicalBERT	0	0.960 (0.955 – 0.964)	0.918 (0.912 – 0.926)	0.989 (0.986 – 0.991)	0.964 (0.960 – 0.969)	0.981 (0.978 – 0.984)	0.981 (0.977 – 0.984)	0.960 (0.955 – 0.965)	0.973 (0.967 – 0.978)	0.966 (0.964 – 0.967)
bert-base-uncased	0	0.957 (0.953 – 0.963)	0.917 (0.909 – 0.924)	0.989 (0.986 – 0.991)	0.964 (0.959 – 0.968)	0.982 (0.977 – 0.985)	0.981 (0.978 – 0.985)	0.984 (0.981 – 0.988)	0.974 (0.968 – 0.980)	0.969 (0.967 – 0.971)
biobert-v1.1	0	0.959 (0.954 – 0.963)	0.918 (0.912 – 0.924)	0.989 (0.987 – 0.991)	0.964 (0.960 – 0.968)	0.981 (0.978 – 0.984)	0.981 (0.978 – 0.984)	0.987 (0.983 – 0.990)	0.977 (0.973 – 0.982)	0.969 (0.968 – 0.971)
ClinicalBERT	0	0.986 (0.983 – 0.989)	0.917 (0.911 – 0.924)	0.989 (0.986 – 0.991)	0.964 (0.959 – 0.968)	0.981 (0.977 – 0.983)	0.983 (0.980 – 0.986)	0.978 (0.974 – 0.983)	0.968 (0.963 – 0.975)	0.971 (0.969 – 0.972)
DeepSeek-R1-Distill-Llama-8B	10	0.996 (0.994 – 0.997)	0.995 (0.993 – 0.997)	0.999 (0.998 – 1.000)	0.990 (0.987 – 0.992)	0.992 (0.989 – 0.994)	0.997 (0.995 – 0.998)	0.969 (0.965 – 0.973)	0.845 (0.834 – 0.859)	0.973 (0.971 – 0.975)
o3-mini-2025-01-31	10	0.997 (0.995 – 0.998)	0.998 (0.997 – 0.999)	1.000 (0.999 – 1.000)	0.988 (0.986 – 0.990)	0.994 (0.992 – 0.996)	**1.000 (0.999 – 1.000)**	0.997 (0.995 – 0.998)	0.923 (0.914 – 0.931)	0.987 (0.986 – 0.988)
Llama3-Med42–8B	10	0.997 (0.996 – 0.999)	0.999 (0.998 – 1.000)	**1.000 (1.000 – 1.000)**	0.998 (0.997 – 0.999)	0.999 (0.998 – 1.000)	0.999 (0.998 – 1.000)	0.996 (0.994 – 0.997)	0.914 (0.905 – 0.924)	0.988 (0.987 – 0.989)
gpt-3.5-turbo-0125	10	0.994 (0.992 – 0.996)	0.998 (0.997 – 0.999)	1.000 (0.999 – 1.000)	0.998 (0.997 – 0.999)	0.996 (0.995 – 0.997)	0.999 (0.998 – 1.000)	0.991 (0.988 – 0.993)	0.938 (0.930 – 0.947)	0.989 (0.988 – 0.990)
Llama-3.1–8B-Instruct	10	0.995 (0.994 – 0.997)	0.997 (0.996 – 0.998)	0.998 (0.997 – 0.999)	0.995 (0.994 – 0.997)	**0.999 (0.999 – 1.000)**	0.999 (0.999 – 1.000)	0.996 (0.994 – 0.997)	0.934 (0.927 – 0.942)	0.989 (0.988 – 0.991)
DeepSeek-R1-Distill-Llama-70B	0	0.998 (0.997 – 0.999)	0.999 (0.998 – 1.000)	0.999 (0.998 – 0.999)	0.999 (0.997 – 0.999)	0.999 (0.998 – 1.000)	**1.000 (0.999 – 1.000)**	0.998 (0.996 – 0.999)	0.934 (0.925 – 0.942)	0.991 (0.990 – 0.992)
gpt-4o-2024-08-06	3	0.995 (0.993 – 0.997)	0.998 (0.997 – 0.999)	**1.000 (1.000 – 1.000)**	0.998 (0.997 – 0.999)	0.999 (0.998 – 1.000)	0.999 (0.999 – 1.000)	0.997 (0.995 – 0.998)	0.952 (0.944 – 0.959)	0.992 (0.991 – 0.993)
Llama-DrugDetector-70B	0	0.998 (0.997 – 0.999)	0.999 (0.998 – 0.999)	0.999 (0.998 – 0.999)	0.998 (0.997 – 0.999)	0.999 (0.997 – 0.999)	**1.000 (0.999 – 1.000)**	0.998 (0.997 – 0.999)	0.959 (0.953 – 0.966)	0.994 (0.993 – 0.995)
Llama-DrugDetector-8B	0	0.998 (0.998 – 0.999)	0.999 (0.998 – 1.000)	0.998 (0.997 – 0.999)	0.999 (0.998 – 1.000)	0.986 (0.983 – 0.989)	0.999 (0.999 – 1.000)	0.995 (0.993 – 0.997)	0.982 (0.977 – 0.987)	0.995 (0.994 – 0.995)
MedLlama3-8B	10	0.999 (0.997 – 1.000)	0.999 (0.998 – 1.000)	0.999 (0.999 – 1.000)	0.997 (0.996 – 0.998)	0.996 (0.994 – 0.998)	0.999 (0.998 – 0.999)	0.996 (0.993 – 0.999)	0.980 (0.961 – 0.991)	0.996 (0.993 – 0.997)
Llama-3.1–70B-Instruct	0	**0.999 (0.998 – 1.000)**	0.999 (0.998 – 1.000)	0.999 (0.998 – 1.000)	1.000 (0.999 – 1.000)	**0.999 (0.999 – 1.000)**	**1.000 (0.999 – 1.000)**	0.999 (0.998 – 1.000)	0.985 (0.980 – 0.988)	0.997 (0.997 – 0.998)
Llama-3.3–70B-Instruct	0	**0.999 (0.998 – 1.000)**	1.000 (0.999 – 1.000)	1.000 (0.999 – 1.000)	**1.000 (1.000 – 1.000)**	**0.999 (0.999 – 1.000)**	**1.000 (0.999 – 1.000)**	**0.999 (0.999 – 1.000)**	0.982 (0.978 – 0.987)	0.997 (0.997 – 0.998)
Llama3-Med42–70B	10	0.998 (0.997 – 0.999)	0.999 (0.999 – 1.000)	**1.000 (1.000 – 1.000)**	1.000 (0.999 – 1.000)	**0.999 (0.999 – 1.000)**	**1.000 (0.999 – 1.000)**	**1.000 (0.999 – 1.000)**	0.990 (0.986 – 0.993)	0.998 (0.998 – 0.999)
Llama3-0penBioLLM-70B	5	0.999 (0.998 – 0.999)	**1.000 (1.000 – 1.000)**	**1.000 (1.000 – 1.000)**	0.999 (0.999 – 1.000)	**0.999 (0.999 – 1.000)**	**1.000 (0.999 – 1.000)**	**1.000 (0.999 – 1.000)**	**0.994 (0.991 – 0.996)**	**0.999 (0.998 – 0.999)**

**Table F.13:** Mean Specificity on the held-out test set (n=500) with bootstrapped lower and upper bounds for each detector and drug class. Abbreviations: Methamphetamine (Meth.), Benzodiazepine (Benzo.), Prescription Opioid Misuse (Rx. Opioids), Injection Drug Use (IDU).

Detector	Shots	Heroin	Cocaine	Meth.	Benzos.	Rx. Opioids	Cannabis	IDU	Any	Overall
MedLlama3-8B	5	0.257 (0.246 – 0.266)	0.841 (0.832 – 0.850)	0.935 (0.929 – 0.943)	0.935 (0.930 – 0.941)	0.834 (0.825 – 0.843)	0.876 (0.867 – 0.886)	0.348 (0.334 – 0.360)	0.195 (0.182 – 0.206)	0.653 (0.647 – 0.659)
Llama3-Med42–70B	0	0.842 (0.831 – 0.851)	0.946 (0.942 – 0.951)	0.975 (0.972 – 0.979)	0.966 (0.961 – 0.969)	0.905 (0.897 – 0.911)	0.958 (0.953 – 0.962)	0.785 (0.775 – 0.797)	0.886 (0.877 – 0.896)	0.908 (0.904 – 0.912)
Llama3-Med42–8B	10	0.807 (0.797 – 0.820)	0.952 (0.947 – 0.958)	0.969 (0.965 – 0.974)	0.959 (0.954 – 0.963)	0.910 (0.904 – 0.917)	0.970 (0.967 – 0.975)	0.926 (0.918 – 0.933)	0.969 (0.964 – 0.976)	0.933 (0.929 – 0.938)
Llama3-0penBioLLM-70B	10	0.871 (0.861 – 0.881)	0.979 (0.974 – 0.982)	0.983 (0.980 – 0.986)	0.971 (0.966 – 0.975)	0.909 (0.903 – 0.917)	0.985 (0.982 – 0.988)	0.956 (0.951 – 0.962)	0.944 (0.938 – 0.952)	0.950 (0.947 – 0.953)
biobert-v1.1	0	0.919 (0.913 – 0.926)	0.997 (0.996 – 0.998)	0.999 (0.999 – 1.000)	**0.998 (0.996 – 0.999)**	0.999 (0.998 – 1.000)	0.999 (0.998 – 1.000)	0.805 (0.795 – 0.814)	0.915 (0.908 – 0.923)	0.954 (0.952 – 0.956)
ClinicalBERT	0	0.928 (0.922 – 0.934)	0.994 (0.992 – 0.995)	0.996 (0.995 – 0.998)	0.998 (0.997 – 0.999)	0.998 (0.997 – 0.999)	0.991 (0.989 – 0.993)	0.810 (0.800 – 0.818)	0.920 (0.911 – 0.929)	0.954 (0.952 – 0.957)
Llama-3.1–8B-Instruct	0	0.930 (0.923 – 0.937)	0.986 (0.984 – 0.988)	0.977 (0.974 – 0.981)	0.975 (0.971 – 0.979)	0.873 (0.865 – 0.881)	0.992 (0.990 – 0.994)	0.929 (0.923 – 0.936)	0.993 (0.990 – 0.995)	0.957 (0.955 – 0.959)
bert-base-uncased	0	0.962 (0.957 – 0.966)	0.998 (0.997 – 0.999)	**1.000 (0.999 – 1.000)**	0.997 (0.996 – 0.999)	0.998 (0.997 – 0.999)	0.999 (0.998 – 1.000)	0.810 (0.800 – 0.820)	0.930 (0.921 – 0.939)	0.962 (0.959 – 0.964)
Bio_ClinicalBERT	0	0.942 (0.936 – 0.948)	0.997 (0.995 – 0.998)	0.999 (0.999 – 1.000)	0.995 (0.993 – 0.996)	0.997 (0.996 – 0.998)	0.997 (0.996 – 0.998)	0.861 (0.851 – 0.870)	0.905 (0.896 – 0.914)	0.962 (0.959 – 0.964)
Llama-3.3–70B-Instruct	0	0.910 (0.902 – 0.916)	0.997 (0.996 – 0.998)	0.997 (0.996 – 0.998)	0.990 (0.988 – 0.993)	0.926 (0.920 – 0.932)	0.997 (0.996 – 0.998)	0.944 (0.938 – 0.951)	0.977 (0.973 – 0.983)	0.967 (0.966 – 0.969)
Llama-3.1–70B-Instruct	0	0.943 (0.937 – 0.950)	0.998 (0.997 – 0.999)	0.998 (0.997 – 0.999)	0.989 (0.986 – 0.991)	0.929 (0.924 – 0.935)	0.998 (0.997 – 0.999)	0.949 (0.944 – 0.955)	0.969 (0.963 – 0.975)	0.972 (0.970 – 0.974)
gpt-3.5-turbo-0125	10	0.940 (0.934 – 0.947)	0.992 (0.990 – 0.994)	0.987 (0.984 – 0.990)	0.979 (0.976 – 0.983)	0.965 (0.960 – 0.969)	0.997 (0.995 – 0.998)	0.958 (0.952 – 0.964)	0.964 (0.959 – 0.970)	0.973 (0.971 – 0.975)
DeepSeek-R1-Distill-Llama-8B	5	0.940 (0.934 – 0.945)	0.982 (0.978 – 0.985)	0.988 (0.986 – 0.991)	0.987 (0.984 – 0.990)	0.978 (0.975 – 0.982)	0.991 (0.988 – 0.993)	0.959 (0.953 – 0.963)	0.962 (0.957 – 0.968)	0.974 (0.971 – 0.976)
DeepSeek-R1-Distill-Llama-70B	5	0.935 (0.929 – 0.942)	0.995 (0.993 – 0.996)	0.996 (0.994 – 0.997)	0.998 (0.997 – 0.999)	0.972 (0.968 – 0.976)	0.999 (0.998 – 0.999)	0.993 (0.991 – 0.995)	0.995 (0.993 – 0.997)	0.985 (0.984 – 0.987)
gpt-4o-2024-08-06	0	0.987 (0.984 – 0.990)	0.999 (0.998 – 1.000)	0.999 (0.998 – 1.000)	0.996 (0.995 – 0.998)	0.950 (0.945 – 0.954)	0.999 (0.998 – 1.000)	0.994 (0.992 – 0.996)	0.991 (0.987 – 0.993)	0.989 (0.988 – 0.990)
Llama3-0penBioLLM-8B	10	0.978 (0.974 – 0.981)	0.997 (0.995 – 0.998)	0.996 (0.994 – 0.997)	0.995 (0.994 – 0.997)	0.985 (0.982 – 0.987)	0.999 (0.999 – 1.000)	0.982 (0.978 – 0.985)	0.987 (0.982 – 0.990)	0.990 (0.989 – 0.991)
Llama-DrugDetector-70B	0	0.973 (0.969 – 0.977)	0.999 (0.999 – 1.000)	**1.000 (0.999 – 1.000)**	0.997 (0.996 – 0.999)	0.981 (0.978 – 0.985)	**1.000 (0.999 – 1.000)**	0.996 (0.994 – 0.997)	**0.997 (0.995 – 0.999)**	0.993 (0.992 – 0.994)
Llama-DrugDetector-8B	3	0.993 (0.991 – 0.995)	0.999 (0.998 – 0.999)	0.999 (0.998 – 0.999)	0.992 (0.990 – 0.994)	0.996 (0.995 – 0.998)	0.999 (0.998 – 1.000)	0.997 (0.996 – 0.999)	0.978 (0.973 – 0.982)	0.994 (0.993 – 0.995)
o3-mini-2025-01-31	10	**0.996 (0.994 – 0.997)**	**1.000 (0.999 – 1.000)**	0.999 (0.998 – 1.000)	**0.999 (0.998 – 1.000)**	0.973 (0.969 – 0.976)	0.999 (0.999 – 1.000)	**0.998 (0.997 – 0.999)**	0.992 (0.988 – 0.995)	**0.994 (0.994 – 0.995)**
